# Mutational Analysis of Sclerostin Shows Importance of the Flexible Loop and the Cystine-Knot for Wnt-Signaling Inhibition

**DOI:** 10.1371/journal.pone.0081710

**Published:** 2013-11-29

**Authors:** Verena Boschert, Maarten van Dinther, Stella Weidauer, Katharina van Pee, Eva-Maria Muth, Peter ten Dijke, Thomas D. Mueller

**Affiliations:** 1 Department of Molecular Plant Physiology and Biophysics, Julius-von-Sachs Institute of the University Wuerzburg, Wuerzburg, Germany; 2 Department of Molecular Cell Biology, Cancer Genomics Center Netherlands and Center for Biomedical Genetics, Leiden University Medical Center, Leiden, The Netherlands; Tohoku University, Japan

## Abstract

The cystine-knot containing protein Sclerostin is an important negative regulator of bone growth and therefore represents a promising therapeutic target. It exerts its biological task by inhibiting the Wnt (wingless and int1) signaling pathway, which participates in bone formation by promoting the differentiation of mesenchymal stem cells to osteoblasts. The core structure of Sclerostin consists of three loops with the first and third loop (Finger 1 and Finger 2) forming a structured β-sheet and the second loop being unstructured and highly flexible. Biochemical data showed that the flexible loop is important for binding of Sclerostin to Wnt co-receptors of the low-density lipoprotein related-protein family (LRP), by interacting with the Wnt co-receptors LRP5 or -6 it inhibits Wnt signaling. To further examine the structural requirements for Wnt inhibition, we performed an extensive mutational study within all three loops of the Sclerostin core domain involving single and multiple mutations as well as truncation of important regions. By this approach we could confirm the importance of the second loop and especially of amino acids Asn92 and Ile94 for binding to LRP6. Based on a Sclerostin variant found in a Turkish family suffering from Sclerosteosis we generated a Sclerostin mutant with cysteines 84 and 142 exchanged thereby removing the third disulfide bond of the cystine-knot. This mutant binds to LRP6 with reduced binding affinity and also exhibits a strongly reduced inhibitory activity against Wnt1 thereby showing that also elements outside the flexible loop are important for inhibition of Wnt by Sclerostin. Additionally, we examined the effect of the mutations on the inhibition of two different Wnt proteins, Wnt3a and Wnt1. We could detect clear differences in the inhibition of these proteins, suggesting that the mechanism by which Sclerostin antagonizes Wnt1 and Wnt3a is fundamentally different.

## Introduction

The human skeleton has about 200 bones forming a very complex tissue with a multitude of functions. It stabilizes and protects the inner organs but on the other hand also serves as a storage pool for the important ions calcium and phosphate. In the bone marrow important hematopoietic cells such as the erythrocytes, the thrombocytes or the T- and B-lymphocytes are formed. Although bone seems unchanging at first sight, it is not a dead tissue, but goes through permanent life-long modeling and remodeling processes not only during upgrowth but also after its ending i.e. the 2^nd^ decade in life. Damages in bone caused by mechanical stress are continuously repaired; hormones regulate the release of calcium and phosphate to replenish blood serum level by decomposing bones [1]. To execute these tasks a tightly regulated system of interacting cells is required. Among these are the “bone-forming” osteoblasts, which buildup the osteoid by secretion of extracellular matrix [2]. After mineralization, the osteoblasts differentiate to osteocytes, the master regulators of bone growth and depletion. A third cell type is the osteoclast, which is the opponent of the osteoblast and thus is responsible for dismantling the bone tissue [3].

For the development of the osteoblasts the Bone Morphogenetic Proteins (BMP) signaling pathway plays an important role [4]. Besides the former also the canonical Wnt (Wingless and Int1) signaling pathway has been shown to contribute to bone formation. The signaling strength of the Wnt pathway determines whether mesenchymal stem cells (MSC) differentiate to either chondrocytes or osteoblasts with a weak Wnt signal leading to the formation of chondrocytes and strong Wnt activity resulting in differentiation to osteoblasts [5]. Deregulation of bone formation and resorption leads to severe diseases. Defects in bone resorption due to lower activity in osteoclasts result in osteopetrosis with patients showing increased bone mass [6]. On the contrary increased activity in osteoclasts or decreased activity in osteoblasts leads to a reduction in bone mass. The most prominent disease showing decreased bone mass is osteoporosis, which affects more than 30% of women after menopause [7]. The molecular cause is loss of estrogen leading to the increased expression of tumor necrosis factor (TNF)α, interleukin (IL)-1, macrophage-colony stimulating factor (M-CSF) and receptor activator on nuclear factor κ-B ligand (RANKL) all of which are positive regulators of osteoclastogenesis [8], [9]. Most current therapeutics target at osteoclast activity via anti-catabolic mechanisms thereby preventing further loss of bone mass [10]. The only common osteoanabolic option is the application of parathyroid hormone (PTH), an 84 amino acids (aa) peptide hormone, which increases the number of osteoblasts [11]. However, a very stringent application scheme is required and PTH overproduction (or application) reverses its osteoanabolic function [12]. In rats overdosing of PTH has even led to the formation of osteosarcoma and thus application of PTH in humans is currently limited to two years [13]. This shows the strong need for an alternative osteoanabolic therapy to efficiently target osteoporosis.

Sclerostin, originally identified in genetic screens of two diseases characterized by strong increased bone mass, Sclerosteosis and van Buchem syndrome, could provide such a target. Here gene expression of SOST (encoding Sclerostin) is either lost or blunted indicating that Sclerostin is a negative regulator of bone formation and its inhibition might restore normal bone formation rate [14], [15]. Interestingly, small nuclear polymorphism in the regulator elements of the SOST gene could be linked to predisposition for osteoporosis [16]. Initially, Sclerostin was described as a direct high-affinity antagonist of BMP signaling belonging to the DAN family of BMP modulator proteins [17], [18]. It is a secreted 213aa glycoprotein containing a consensus motif for a cystine-knot, but outside the cystine-knot motif Sclerostin shares very little sequence homology with the other DAN family members. The highly bone-specific phenotype of SOST loss-of-function mutants might be explained by the fact that osteocytes are the sole source of Sclerostin [19], [20]. Most importantly, Sclerostin seems not to affect osteoclasts but rather limits the activity of osteoblasts by impeding proliferation and differentiation of mesenchymal stem cells (MSCs) to osteoblasts, as well as decreasing mineralization and inducing apoptosis in osteoblasts [18], [21], [22]. Although these properties make Sclerostin a prime target for an osteoanabolic therapy approach against osteoporosis, little is known about its molecular mechanism of action. In contrast to previous reports Sclerostin is a direct modulator/inhibitor of the Wnt signaling pathway and not an antagonist of extracellular BMPs [23]. Its BMP-inhibiting activities were shown to rather occur in a cell autonomous manner by sequestering BMP inside the cell [24]. Like the classical Wnt inhibitor Dickkopf1 (Dkk1) Sclerostin binds to the Wnt co-receptor LRP5 or the homologue LRP6 and thereby antagonizes Wnt activity [25]. Deletion studies showed that the binding site on LRP5 resides in the first two of the four YWTD β-propeller domains present in the ectodomain of LRP5 [26]. New findings revealed that for binding to the first LRP6 β-propeller domains a NXI amino acid motif common in Wnt-inhibitors like Sclerostin and Dkk1 is crucial [27]. By performing an extensive mutational study on the structured part of Sclerostin we could confirm the importance of the loop including this sequence motif. Furthermore we could show that apart from this part of the protein also the cystine-knot is important for Sclerostin activity.

## Materials and Methods

### Expression of Sclerostin in E.coli

Wildtype and variant proteins of full-length murine Sclerostin (Sclerostin) were obtained from bacterial expression as described previously [28]. All proteins were purified by a two-step chromatography with first performing a cation-exchange chromatography and a subsequent reversed-phase HPLC employing a 0.1% trifluoroacetic acid/acetonitrile gradient to produce homogeneous and pure Sclerostin protein. Recombinant proteins were analysed by SDS-PAGE and ESI-FT ICR mass spectrometry confirming the theoretical mass and ensuring a purity of greater than 95%.

Single and double mutants of Sclerostin were generated by site directed mutagenesis using the Rapid PCR methodology by Weiner and Costa [29]. For the generation of deletion variants (Sclerostin ΔLoop) or Sclerostin variants containing multiple mutations (Sclerostin Alaloop, Sclerostin F1mut, and Sclerostin F2mut) cDNAs containing all mutations were obtained by gene synthesis (Geneart). Expression, refolding and purification of the Sclerostin variants followed the process described for wildtype murine Sclerostin [28].

Absorbance of purified wildtype and mutant Sclerostin proteins at 280 nm was measured and protein concentration was determined by using extinction factors that were calculated from the amino acid sequence of the proteins using the software ProtParam (http://web.expasy.org/protparam; SIB Swiss institute of bioinformatics).

Production of Sclerostin and hLRP6 E1E2 in Freestyle™293F-cells.

The cDNAs encoding for the first two propeller domains of the human LRP6 (termed LRP6E1E2) as well as for murine Sclerostin and variants thereof were cloned into the expression vector pHLsec [30] using the restriction sites for the endonucleases *Age*I and *Kpn*I. The resulting protein constructs consist of an N-terminal signal peptide for secretion of the protein into the medium and a C-terminal hexahistidine sequence for metal ion affinity chromatography. Freestyle™293F-cells were cultivated according to manufacturer's recommendation. For transfection in 6-well plates (Sclerostin and variants thereof) or in 2 L flasks (hLRP6E1E2) cells were grown to a density of 8.5×10^5^ cells/ml. 5 µg DNA and 2 µl PEI (25 kDa, linear, 1 mg/ml) were used in the transfection reaction per ml cell suspension. Additionally, for expression of hLRP6E1E2 2.5 µg vector DNA encoding for the chaperone MESD were added per ml cell suspension. The PEI-DNA complex formation was performed in OptiMEM for 20 min at room temperature. For purification of LRP6E1E2 cell supernatant was supplemented with 300 mM NaCl and loaded onto a 1 ml His-Trap™ excel column (GE Healthcare). The resin was washed with 40 ml buffer (20 mM Tris-HCl, 300 mM NaCl, 20 mM imidazole, pH 8.0) and then the protein was eluted in 1 ml fractions (20 mM Tris-HCl, 300 mM NaCl, 500 mM imidazole pH 8.0). Buffer was exchanged for HBS150 (10 mM Hepes, 150 mM NaCl) by dialysis, the protein was concentrated by ultrafiltration and supplemented with 50% glycerol for storage at 4°C. The protein concentration was determined as described above. The Sclerostin proteins derived from eukaryotic cell culture were used without purification as conditioned medium of the cell culture supernatant. The concentration of Sclerostin in these supernatants was determined by Western blot analysis using anti-His-tag antibodies and comparing the intensities of the protein bands to those of a concentration series of purified E. coli derived Sclerostin protein. For a more accurate estimate of the concentration, the blots were scanned and the intensities of the bands were quantified using the software ImageJ.

Luciferase transcriptional reporter gene assay for measuring Wnt/β-Catenin signaling.

Cells were cultivated at 37°C and 5% CO_2_ in DMEM (Invitrogen) containing 10% (v/v) FCS, 100 U/ml penicillin G, 100 µg/ml streptomycin (Invitrogen). For generation of HEK-293TSA M50, a stable reporter cell line for quantitative measurement of canonical Wnt/β-Catenin activity, HEK-293TSA cells were seeded in a 6-well plate at a density of 4×10^5^ cells/ml. After 24 h cells were transfected with 1.8 µg of M50 Super 8xTopFlash (provided by Randall Moon) and 200 ng linearized hygromycin marker DNA (Clontech) using HEKFectin (Bio-Rad) according to the manufacturer's protocol. Two days after transfection cells were transferred to a 6 cm culture plate (Greiner) and selection of clones was initiated by adding 200 µg/ml hygromycin. Selection was continued for several rounds and different pools of stably transfected cells were tested. A cell pool, which showed a dose-dependent response of the reporter to recombinant Wnt3a was then selected for further reporter gene analysis.

For measurement of Wnt3a activity HEK-293TSA M50 cells were seeded in a 96-well plate at a density of 2.5×10^5^ cells/ml. After 24 h the cells were stimulated using 1.5 nM mWnt3a (R&D Systems) in the presence of varying concentrations (1 µM-0.2 µM) of wildtype Sclerostin or variants thereof. The stimulated cells were lysed after 24 h using 50 µl reporter lysis buffer (Promega) per well and a single freeze and thaw cycle at −80°C. 20 µl of the cell lysate were mixed with 50 µl of luciferase assay substrate (Promega). Luciferase activity was measured using a luminometer (Luminoscan Ascent, Labsystems). Data analysis was performed with Prism 5 (Graphpad) using nonlinear regression.

For measurement of Wnt1 activity 15 ml HEK-293TSA M50 cell suspension was seeded in 10 cm culture dishes at a density of 1.5×10^5^ cells/ml. After 24 h the cells were transfected with 12 µg of an expression vector for mWnt1 (mouse cDNA clone MC205633, Origene) or 12 µg empty vector (pEF6B, Invitrogen) per well using 0.3 µl Xfect reagent/µg DNA (Clontech). On the following day cells were seeded to 80% confluency in 96-well plates. 48 hours after transfection cells were stimulated with different concentrations of wildtype Sclerostin or mutants thereof. Stimulated cells were lysed after 24 h using 50 µl reporter lysis buffer (Promega) per well and a single freeze and thaw cycle at −80°C. Luciferase activities were measured as stated above.

### Cellular β-Catenin-Assay

The assay is based on the publication of Hannoush, 2008 [31]. HEK293TSA M50 cells were seeded at a density of 2×10^4^ per well into a D-lysine coated, white, clear bottom 96-well plate (Greiner). After 12 h cells were stimulated with 4.4 nM recombinant mWnt3a and different concentrations of Sclerostin (4-0.03 µM) or mDkk1 (20-0.2 nM, R&D Systems). As a positive control, cells were treated with 50, 25 and 12.5 mM LiCl. After 6 h cells were fixed with 4% paraformaldehyde in medium, washed three times with PBS and permeabilized 3 times with PBS containing 0.1% Triton X-100 for 2 minutes. Blocking was performed over night with Licor blocking buffer MB-070 (Rockland). On the next day, after washing with PBS/0.1% Tween 20, cells were incubated for 1 h with an antibody specific for β-Catenin (cat# 610154, Beckton Dickinson) diluted 1∶200 in blocking buffer. The plate was incubated for 1 h with a secondary antibody (1∶200, anti-mouse IRDye 800 CW, Rockland) and Draq5 (0.5 nM, Biostatus). After washing, buffer was removed and the plate was scanned at 700 and 800 nm (Odyssey, Licor). Data analysis was performed as described [31].

For analysis upon Wnt1 stimulation, 2×10^6^ HEK293TSA M50 cells were seeded in 15 ml medium in 10 cm cell culture dishes and transfected with 12 µg pCMV6-Kan/Neo expression vector encoding for mWnt1 (mouse cDNA clone MC205633, Origene) or empty vector (pEF6B, Invitrogen) using 3.6 µl Xfect reagent (Clontech). After 12 h incubation the cells were seeded to 90% confluency in 96-well plates. 24 h later cells were treated with different concentrations of Sclerostin (133-0.5 nM) and mDkk1 (13-0.05 nM, R&D Systems) for 4 h. Cells transfected with empty vector were treated with 33, 17 and 8 mM LiCl. Immunodetection, staining and data analysis were performed as indicated above.

### Radioligand binding assay for binding of Sclerostin to LRP6

Iodination of proteins followed a protocol adapted from the method by Frolik et al. [32]. For iodination of Sclerostin or the variants 1 µg protein was dissolved in 13 µl 2 M sodium phosphate buffer pH 7.5. The protein was incubated with 5 µl of Na^125^I (18MBq, Perkin Elmer) and 5 µl of Chloramine T (100 µg/ml) for 2 minutes. Another 5 µl Chloramine T were added two more times followed by 2 minutes incubation steps each. The reaction was stopped by adding 20 µl N-acetyl tyrosine (50 mM), 20 µl potassium iodide (60 mM) and 200 µl urea (1.2 g in 1 ml water) in 1 M acetic acid and incubation of this mixture for 2 minutes. The proteins were separated from non-reacted iodine using a G25 (PD-10) size exclusion column equilibrated with column buffer (75 mM NaCl, 4 mM HCl, 1 mg/ml BSA). Fractions containing ^125^I-radiolabeled protein were pooled and used immediately or stored at 4°C until further use.

For the crosslinking assay COS1 cells were seeded in 10 cm dishes in DMEM containing 10% (v/v) FCS at 75% confluency and transfected the next day using linear PEI 25 kDa and 10 µg of the LRP6 expression plasmid (derived from pCS2 LRP6-EGFP, gift from C. Niehrs, DKFZ Heidelberg, Germany). Cells were transferred to 6 well plates after 24 h. The next day cells were washed twice with PBS-B (PBS, 0.91mM CaCl_2_, 0.49 mM MgCl_2_) plus 1 mg/ml BSA, before they were incubated on ice for 2 h with 10 µl ^125^I-labeled wildtype or mutant Sclerostin protein (1 nM) in 0.5 ml PBS-B plus 1 mg/ml BSA. Unlabelled protein was added for competition where indicated. After incubation the cells were washed with PBS-B and crosslinking was performed by incubation for 15 minutes in PBS-B on ice using 0.27 mM disuccinimidyl suberate (DSS) and 0.07 mM bis(sulfosuccinimidyl)-suberate (BS^3^). Then cells were washed with detachment buffer (10 mM Tris-HCl, 1 mM EDTA, 10% glycerol, 1 mM phenylmethylsulfonylfluoride (PMSF), pH 7.4) and scraped off in 150 µl detachment buffer. The suspension was centrifuged for 2 min at 18.000×g and cells were lysed by adding 500 µl solubilisation buffer (10 mM Tris-HCl, 125 mM NaCl, 1 mM EDTA, 1% Triton A-100, pH 7.4) containing a protease inhibitors cocktail mix (PMSF, Aprotinin, Leupeptin (Roche) and incubated on ice for 30 minutes. Debris was removed by centrifugation at 18.000×g for 2 min and protein concentration was determined by performing a Lowry assay (DC Protein Assay, Biorad). For quantification of Sclerostin bound to LRP6 equal amounts of protein were loaded on a SDS-PAGE gel and the electrophoresis was performed over night (7 mA, RT). After fixing and drying, the gel was analysed using a Phosphoimager (STORM imaging system, Amersham Biosciences). Bands were quantified using ImageJ.

### Binding analysis of the Sclerostin-LRP6 interaction using a Pull Down assay

Purified Sclerostin proteins were biotinylated using EZ-Link Sulfo-NHS-LC-Biotin (Thermo Scientific) in a molar ratio of 1∶1.5 in PBS for 1 h at room temperature and overnight at 4°C. For performing the pull down 1 µM purified human LRP6 was incubated for 1 hour at 4°C with 225 nM biotinylated Sclerostin wildtype, ΔLoop or C84AC142R in 500 µl HBS300T (20 mM HEPES, 300 mM NaCl, 0.05% Tween-20). The samples were then mixed and incubated for 1 hour with 40 µl Streptavidin Agarose Beads (Novagen). The beads were centrifuged for 2 min at 2000×g, washed three times with 500 µl HBS300T and finally resuspended in 40 µl SDS-sample-buffer and boiled for 5 minutes at 95°C. Samples of the beads together with samples from the initial reaction mixture (input) were subjected to SDS-PAGE and a subsequent Western-Blot using an antibody recognizing the His_6_-tag present in the recombinant Sclerostin and LRP6 proteins (Penta His HRP conjucate, 5-Prime).

### Interaction analysis using Surface Plasmon resonance

Interaction analysis was performed as described elsewhere using the ProteOn™ XPR36 SPR system (BioRad) [33]. HBS150T (10 mM HEPES, 150 mM NaCl, pH 7.5, 0.005% Tween20) was used as running buffer. Sclerostin wildtype or the ΔLoop variant proteins were immobilized on separate flow channels at a density of 600RU on the surface of a ProteOn™ GLC sensor chip (BioRad). All measurements were performed at 25°C. Interaction with the antibody Fab fragment was measured by injecting 6 different concentrations (100, 75, 50, 25, 12.5 and 6.25 nM in HBS150T) for 200 sec using the single shot kinetic setup. Dissociation data was obtained from subsequent perfusion of the biosensor with running buffer for 250 sec. For analysis of the kinetics the data was fitted employing a Langmuir 1∶1 interaction type model using the software ProteOn™ Manager 3.0 (BioRad).

## Results

### Mutational study on Sclerostin

**Figure 1:**
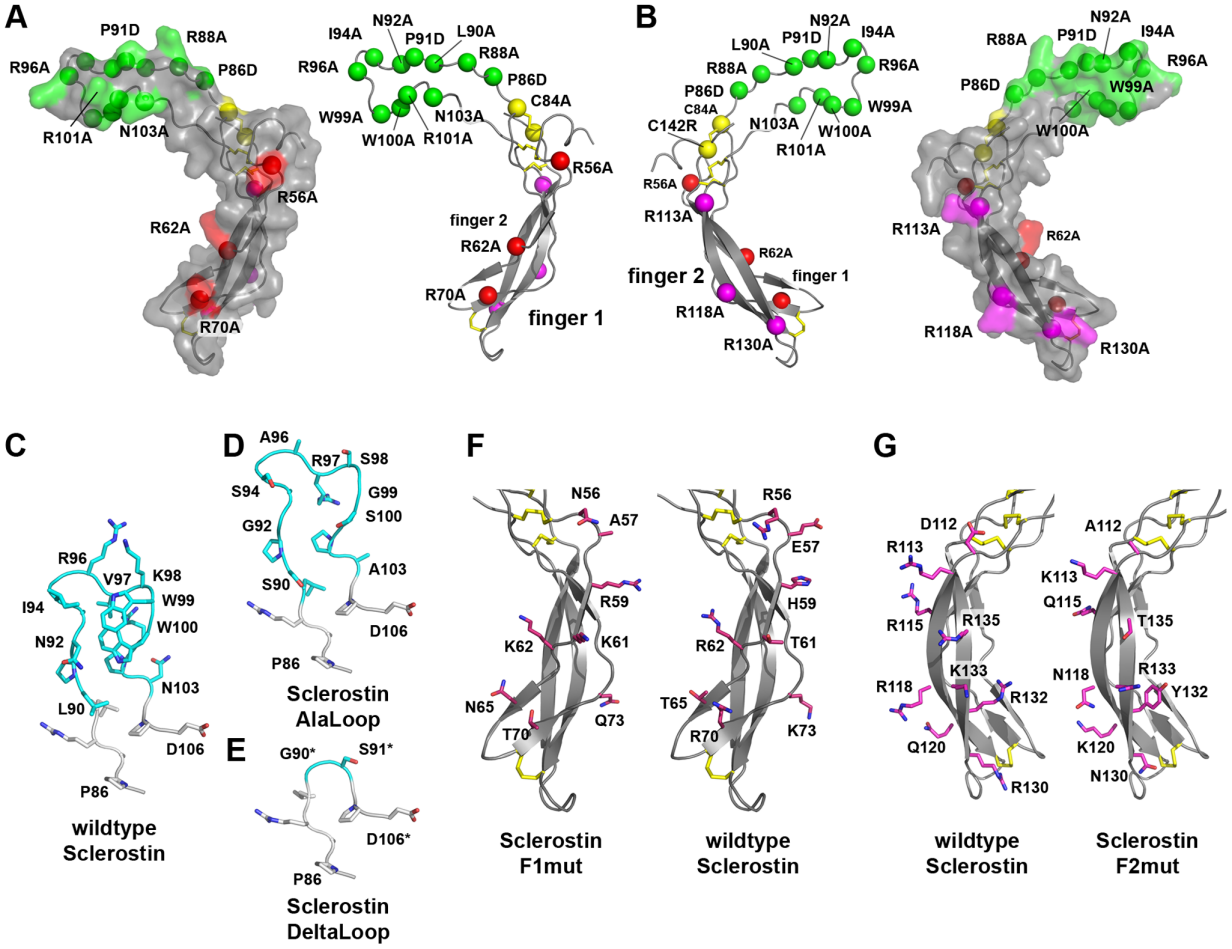
Overview of investigated Sclerostin variants. (A) Surface and secondary structure representation of Sclerostin with residues selected for single point mutations shown as spheres. Residues located in the flexible loop of Sclerostin are colored green, amino acid residues in finger 1 and 2 are indicated in red and magenta, cysteine residues are shown in yellow, respectively. (B) As in (A) but rotated around the y-axis by 180°. (C) Detailed view of the loop region of Sclerostin comprising the residues Gly85 to Asp106. The backbone atoms are shown as ribbon, side chains of non-glycine residues as ball-and-stick models. The region consisting of Leu90 to Asn103 used in the mutational analysis is marked with carbon atoms colored in cyan. (D) Loop region of the Sclerostin multivariant Alaloop. (E) Loop region of the Sclerostin truncation variant ΔLoop, residues Leu90 to Asn103 were replaced by a glycine-serine linker. (F) View of the finger region of wildtype Sclerostin and the Sclerostin multivariant F1mut. Residues shown as ball-and-stick models (carbon atoms colored in red) in finger 1 of wildtype Sclerostin were exchanged to those shown on the left panel. (G) Residues in finger 2 of Sclerostin and their counterpart in the Sclerostin multivariant F2mut are indicated as ball-and-stick models with the carbon atoms colored in magenta.

Previous analyses have shown that the structured cystine-knot motif contains almost completely the Wnt-neutralizing activity of Sclerostin [28], the flexible and disordered N- and C-termini seem not to contribute significantly to inhibition of Wnt3a-mediated reporter gene expression as measured with the TCF/LEF reporter construct M50 8xSuperTOPFlash provided by Randall Moon [34]. We thus focused in our mutagenesis study on positions located within the cystine-knot core but used full-length murine Sclerostin for the analysis. From mutations in the Wnt co-receptor LRP5 found in human patients with a bone-overgrowth phenotype (high-bone mass or HBM) resembling that of the Sclerostin null mutations the Sclerostin binding site was proposed to be localized in the first β-propeller domain of LRP5 [35], [36], [37], [38], [39]. This epitope was later confirmed by deletion studies of LRP5, showing that Sclerostin-mediated Wnt3a inhibition depends on the presence of the first two β-propeller domains [26]. Since both proteins exhibit a highly complimentary charge distribution [28] we opted to mutate first 9 out of 16 arginine residues present in the core region of murine Sclerostin between Asn38 and Arg118 to alanine (Fig. 1A and B). The NMR mapping studies of a Sclerostin-neutralizing antibody reported by Veverka et al. reported for the first time that the Wnt-inhibitor activity of Sclerostin is likely confined in the flexible loop region of Sclerostin [40] (Fig. 1C). A Fab fragment raised against murine Sclerostin, which efficiently neutralizes Sclerostin-activity [41], also binds to the flexible loop region as determined by NMR (Boschert, V., Frisch, C., Schmieder, P., Mueller, T.D., manuscript in preparation) thereby highlighting the importance of the loop region for activity. Conformingly a recent crystallographic study showed a complex of a 7 mer peptide mimicking the Sclerostin loop residues Leu90 to Arg96 bound to propeller 1 of LRP6 [27]. We thus also targeted the flexible loop more specifically and introduced multiple mutations in the loop region of Sclerostin (Fig. 1D and E). For the generation of multi-variants we applied two approaches, first the loop residues from Leu90 to Asn103 of murine Sclerostin were deleted and replaced by a two-residue long linker Gly-Ser (termed ΔLoop, Fig. 1E). The second approach employed multiple mutations in the loop to completely alter the potential binding epitope. Residues Leu90 to Asn103 were therefore exchanged from the original sequence -LPNAIGRVKWWRPN- to -SPGASGARSGSAPA- (termed Alaloop, Fig. 1D). To test that these loop-altering mutations did not affect the fold of murine Sclerostin we tested the binding of Sclerostin ΔLoop to Fab antibodies directed against finger 1 obtained from phage display selection and a directed panning approach using folded peptides mimicking finger 1. These Fab fragments only bind effectively to the oxidatively folded peptide and not to the corresponding linear form. Thus the Fab antibodies recognize a non-linear conformational epitope and can be used as folding indicators for Sclerostin (Back et al., 2012). In vitro interaction analysis shows that affinities and kinetics for the binding of wildtype Sclerostin and the variant ΔLoop to these Fabs are identical (Fig. S1A). These results clearly indicate that mutations in the loop of Sclerostin introduced in the variants ΔLoop and Alaloop did not affect folding of the finger 1/2 core structure. To analyze the effect of individual residue mutation on Sclerostin activity we also performed an alanine scanning approach for most loop residues except for proline 86 and 91, which were substituted for aspartate (Fig. 1A and B, in green).

**Figure 2:**
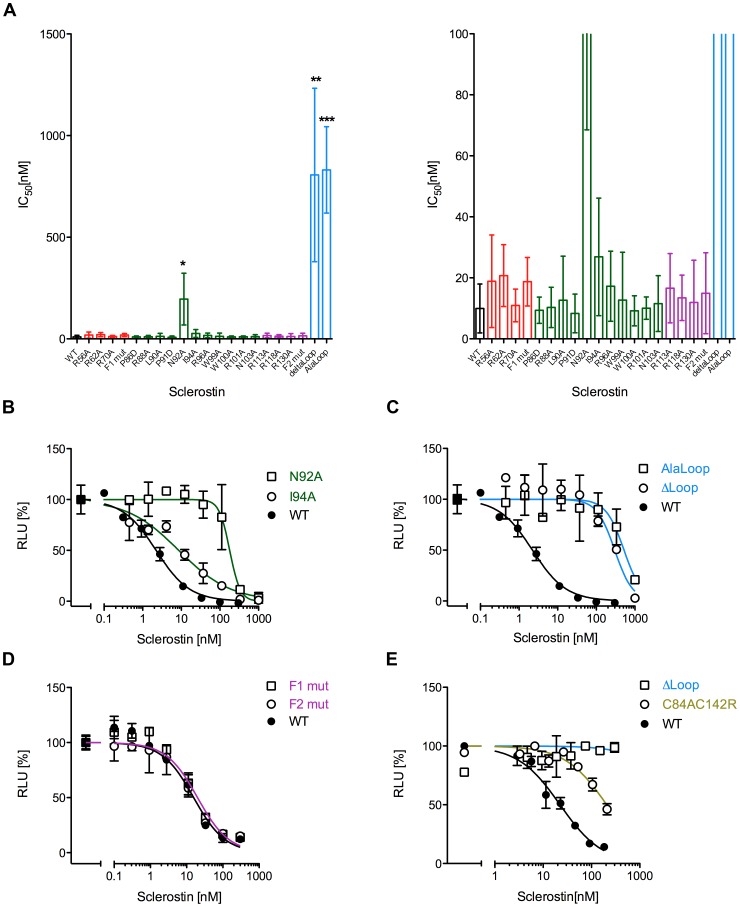
Reporter gene assay to measure the neutralization of mWnt1 activity by wildtype and Sclerostin variants. HEK293TSA cells stably transfected with the Wnt-responsive luciferase reporter construct SuperTOPFlash were transfected with an expression construct for murine Wnt1. After 48 h the cells were stimulated with serial dilutions of wildtype (WT) Sclerostin or variants thereof. (A) Overview of the efficiency of the Sclerostin proteins to neutralize Wnt1 driven luciferase expression (IC_50_ values are shown as bar diagram). The right panel shows a magnification of the data shown in the left panel. Data represents means with standard deviations (SD) of at least three independent experiments. *: P<0.05, **: P<0.01, ***: P<0.001 (student's t-test with data obtained for WT Sclerostin). (B–D) Measurements showing the dose-dependency of selected mutant Sclerostin proteins. IC_50_ values of these experiments are included in the overview shown in (A). Measurements were done in duplicate. (E) Reporter gene assay using supernatants of HEK Freestyle cells expressing Sclerostin mutants C84AC142R, ΔLoop or wildtype Sclerostin. Data points represent duplicates. To highlight the location of the mutation in the Sclerostin structure the same color-coding as in Figure 1 is used.

As no clear picture is yet available for the requirement of residues outside the loop segment of Sclerostin, we similarly used multiple amino acid exchanges combined in a single multi-variant to determine whether elements of finger 1 or finger 2 are involved in Wnt inhibition. For the design of these variants, the structure of murine Sclerostin was used and non-glycine, non-proline residues, which are located at the solvent-accessible surface, were selected for mutagenesis. To avoid refolding problems or decreased solubility, the selected residues were not exchanged uniformly against alanine but rather by an approach in which charged residues were usually substituted for polar but uncharged, and large amino acids were exchanged to smaller ones and *vice versa* unless *in silico* simulations indicated possible folding difficulties. Hence in the variant termed mSOST F1mut eight residues in the Finger1 region (R56N, E57A, H59R, T61K, R62K, T65N, R70T, K73Q, Fig. 2F), in the variant termed mSOST F2mut nine surface-located residues in Finger2 (D112A, R113K, R115Q, R118N, Q120K, R130N, R132Y, K133R, R135T, Fig. 1G) were exchanged accordingly.

A naturally occurring Sclerostin variant, in which residue Cys142 - the sixth cysteine of the cystine-knot - is mutated to arginine, was found in a Turkish family suffering from Sclerosteosis [42]. It was proposed that due to the importance of an intact cystine-knot, the exchange of Cys142 to arginine might disrupt folding of the flexible loop containing the LRP5 binding epitope and thereby (indirectly) lowers Sclerostin neutralization activity. The activity of this mutant has so far only been investigated by analyzing conditioned supernatant of transfected mammalian cells. These experiments, however, show that the mutant protein is only poorly secreted, possibly indicating that the Sclerosteosis in the patients might be due to a Sclerostin dosage effect. To test irrespectively of the cause of Sclerosteosis in these patients whether the cystine-knot is functionally important for Sclerostin activity and LRP6 binding, we first prepared a Sclerostin variant with Cys142 mutated to alanine employing our bacterial expression system. However, by performing mass spectrometry of the purified protein we found that the SH-group of the Cys142 partner Cys84 is not as expected in its free thiol-protonated form but is protected by a glutathione moiety originating from the refolding procedure. As this large tripeptide group attached to Cys84 possibly interferes with Sclerostin activity by an unknown mechanism, we therefore additionally prepared the double variant C84AC142R containing no free SH-group.

### Sclerostin's NXI-motif and parts of the cystine-knot are required for inhibition of Wnt1

Sclerostin-mediated inhibition of Wnt1 activity was determined using a stably transfected reporter cell line developed in house, in which the reporter-driven expression of luciferase is stimulated by coexpression of Wnt1. Using this reporter cell line we determined a concentration for half-maximal Wnt1-inhibition (IC_50_) by wildtype Sclerostin of 10.0±6.9 nM (Fig. 2). Using Wnt1 as a stimulant all arginine-to-alanine variants exhibited IC_50_ values similar to wildtype Sclerostin (Fig. 2A). Deletion or exchange of the loop region (ΔLoop and Alaloop Sclerostin, respectively, Fig. 1C–E), however, resulted in a more than 80-fold increased IC_50_ for the inhibition of Wnt1 (Fig. 2A, C, IC_50_ ΔLoop 806±426 nM, IC_50_ Alaloop 831±213 nM). This finding is consistent data recently reported by Holdsworth et al. [43] and the observation that a 7 mer peptide mimicking the loop residues Leu90 to Arg96 binds to the propeller 1 of LRP5 thereby likely competing for Wnt1 binding [27]. Analysis of the effect of Sclerostin single amino acid mutants on Wnt1 activity also revealed individual residues important for mediating inhibition (Fig. 2A). Two positions important for Sclerostin bioactivity have been recently reported in two other studies [27], [43]. One residue is Ile94, which showed a strong reduction of Sclerostin-mediated Wnt1 inhibition when mutated to non-conserved polar or charged amino acid types [27], [43]. However, when we mutated Ile94 to alanine and thus only removed the hydrophobic side chain functionality, we observed only a very small reduction in bioactivity (Fig. 2A,B) (IC_50_ 26.9±19.3 nM). Similarly, an exchange of Ile94 by slightly smaller valine as reported by Holdsworth et al. resulted in a minor loss in Sclerostin's bioactivity [43]. In our alanine-scanning approach for Sclerostin, the largest effect on Wnt1 inhibition is seen for the variant N92A, whose efficiency to inhibit Wnt1 is decreased nearly 20-fold (IC_50_ 195.7±110.1 nM, compared to 10.0±6.9 nM for wildtype Sclerostin). Holdsworth et al. showed that mutation of Asn92 to any other amino acids disrupts Sclerostin's inhibitory activity, whereas mutation of Ile94 requires a more disrupting non-conservative exchange [43], confirming our analysis that Asn92 presents the hot spot for the Wnt1 inhibitory activity of Sclerostin.

Given the high affinity of Sclerostin for LRP6 [27], the assumption of Asn92 and Ile94 representing the whole epitope of Sclerostin for interacting with LRP5/6 and thereby competing for binding of Wnt1 to LRP6 seemed unreasonable at first sight. We thus investigated whether regions outside the loop also contribute to Sclerostin's activity. However, analysis of both Sclerostin variants containing multiple mutations in the two finger regions shows that the fingers are dispensable for inhibiting Wnt1 (Fig. 2A,D). Both multi-variants F1mut and F2mut showed an inhibitory activity comparable to wildtype Sclerostin with an IC_50_ value of 18.8±6.9 nM for F1mut and an IC_50_ value of 15.0±11.5 nM for F2mut.

**Figure 4:**
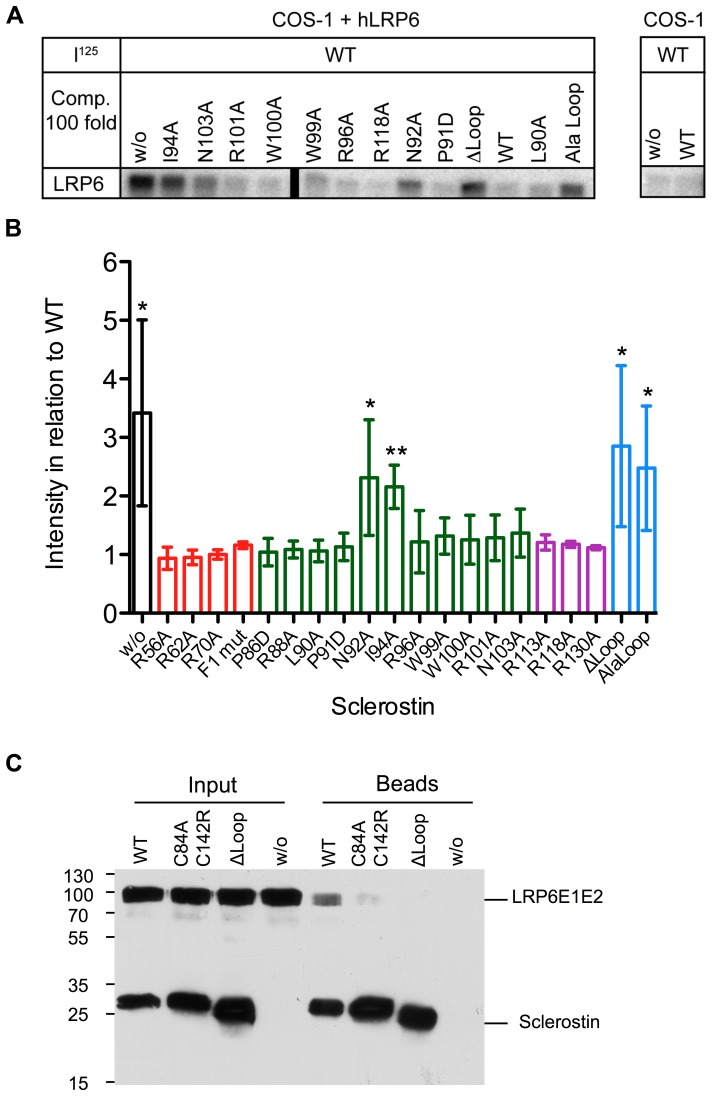
Analysis of binding of wildtype Sclerostin and different variants to hLRP6. (A) COS-1 cells were either transfected with an expression construct encoding for hLRP6 (COS-1 + hLRP6) or mock-transfected using empty vector (COS1). Cells were then incubated with I^125^-labeled wildtype Sclerostin (I^125^). Protein bound to the cell surface was chemically crosslinked in the presence of 2 µg unlabeled wildtype Sclerostin or the indicated Sclerostin variants for competition, corresponding to a 100-fold excess of unlabeled protein. The radioactivity bound to the cells was subsequently analyzed by autoradiography. Shown bands were all obtained in the same experiment. The black bar indicates that the picture was cut to omit samples that are not relevant for this publication. (B) Quantitative autoradiography analysis of several experiments as shown in (A). All bars were normalized by setting the intensity of the protein band obtained from addition of unlabeled wildtype Sclerostin to partially compete off I^125^-labeled wildtype Sclerostin to 1. Data represent means with standard deviations (SD) of at least three independent experiments. *: P<0.05, **: P<0.01 (student's t-test with data obtained for variant R56A). (C) Western Blot of a pull down experiment with biotinylated Sclerostin mutants C84AC142R, ΔLoop and WT Sclerostin. Sclerostin proteins were incubated with purified human LRP6 protein fragment comprising propeller domains E1 and E2 (LRP6E1E2). For pull down of protein complexes streptavidin-agarose beads were used. Input: Samples before beads were added, beads: Beads after incubation and washing. Proteins were detected using an antibody specific for the His_6_-tag of the proteins. One representative out of four experiments is shown.

To find out whether elements of or residues within the cystine-knot are important for inhibition of Wnt1, we expressed a double variant containing the original mutation C142R and C84A. The additional mutation C84A was implemented to avoid low secretion yield as found for the single mutant C142R [42]. Indeed, C84AC142R exhibits a 10-fold decreased efficiency to inhibit Wnt1 activity (Fig. 2E), indicating that mutations outside the NXI-motif can affect Sclerostin activity. Consistent with the attenuated inhibition of Wnt1 seen in the Wnt-reporter assay a decreased binding to the first two propeller of LRP6 (LRP6E1E2) was observed in a pulldown experiment (see also Fig. 4C, Fig. S2B). Although the mutation of Cys142 to arginine and consequently the disruption of the third disulfide bond of the cystine-knot might affect Sclerostin's Wnt1 inhibition indirectly by changing the loop conformation, this is unlikely because of the highly flexible nature of the loop.

### Sclerostin can inhibit Wnt3a by a mechanism different from Wnt1 inhibition

**Figure 3:**
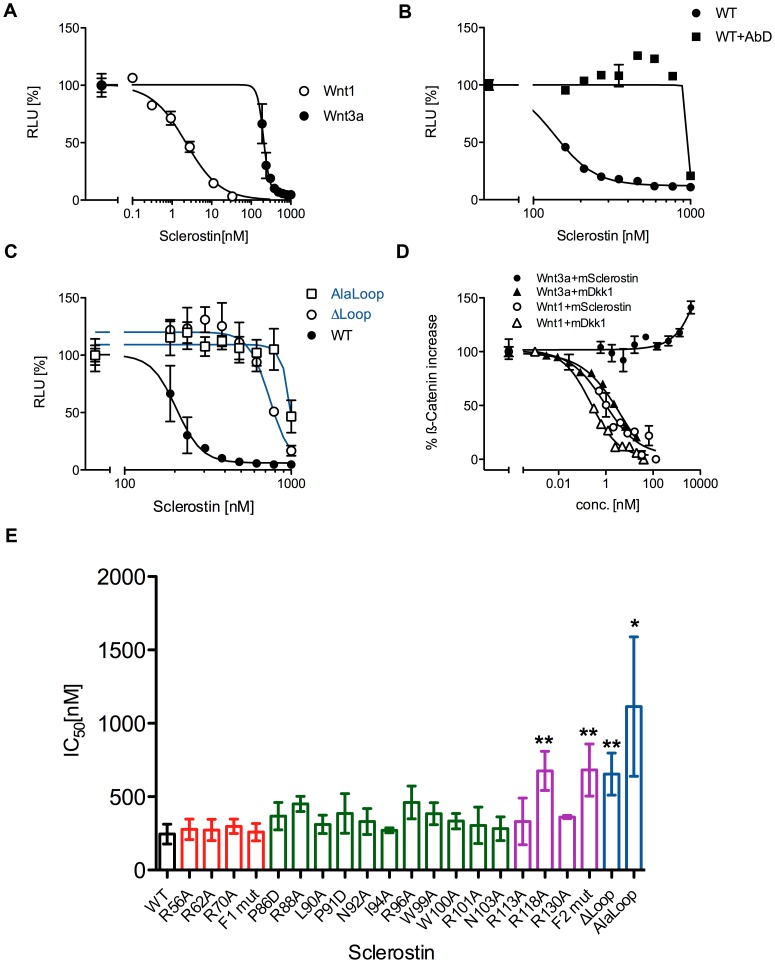
Inhibition of Wnt3a signaling by Sclerostin. (A) HEK293TSA cells stably transfected with the Wnt-responsible luciferase reporter construct SuperTOPFlash were stimulated with 1.5 nM recombinant murine Wnt3a and serial dilutions of murine Sclerostin. Shown is the resulting dose response curve in comparison to the dose response curve of mWnt1-transfected cells as shown in figure 3. (B) mWnt3a-derived luciferase signal obtained in presence of Sclerostin wildtype (WT) and 2 µM Sclerostin specific antibody AbD09097 (AbD) or with WT alone. (C) Reporter gene assay as depicted in (A) and (B) showing the dose-dependency of Sclerostin Alaloop and ΔLoop in comparison to WT Sclerostin. Measurements were done in duplicate. (D) Measurement of the intracellular level of β-Catenin upon mWnt3a and mWnt1 stimulation in presence of Sclerostin or Dkk1 using In-Cell Western. The fluorescence signal determined from the antibody against β-Catenin was normalized for cell number using DNA staining with DRAQ5. Signal of non-transfected/untreated cells was set to 0% and signal of cells treated/transfected with Wnt proteins alone was set to 100%. A typical experiment out of three is shown; datapoints represent means with SD of two independent measurements. (E) Overview of the efficiency of Sclerostin WT and mutant proteins to neutralize Wnt3a driven luciferase expression (IC_50_ values represented as bar diagram). Data represent means with standard deviation (SD) of at least two independent experiments. *: P<0.05 **: P<0.01 (student's t-test with data obtained for wildtype Sclerostin). To highlight the location of the mutation in the Sclerostin structure the same color-coding as in figure 2 is used.

The ability of Sclerostin to inhibit signaling initiated by Wnt3a might be questioned by the finding that Sclerostin and Wnt3a bind to different seemingly non-overlapping regions in the LRP6 receptor ectodomain [26]. Whereas Wnt3a binds to the third and fourth propeller domain, Sclerostin binds, like Wnt1 (and other members of the Wnt1 class), to the first and second propeller domains [44]. Therefore according to this data a competitive inhibition mechanism should only be possible between Wnt1 and Sclerostin, as both proteins bind to the same or at least a highly overlapping site at LRP6. For Wnt3a and Sclerostin Li et al. and Bourhis et al. could show that Sclerostin and Wnt3a can simultaneously bind to LRP6 without affecting their interaction with LRP6 making a competitive inhibition mechanism implausible [26], [27]. Several reports nevertheless showed that Sclerostin could attenuate Wnt3a activity [24], [26], [28], [40], [45], although the required concentrations to efficiently antagonize Wnt3a question the physiological relevance of Sclerostin as Wnt3a inhibitor. Our data and others [40] shows that inhibition of Wnt3a by recombinant Sclerostin (IC_50_ 246±67 nM) requires a more than 100-fold higher Sclerostin concentration compared to that necessary for half-maximal inhibition of Wnt1 (Fig. 3A). Furthermore the Hill coefficient of the dose-dependent inhibition of Wnt3a by Sclerostin clearly deviates from the expected slope being a further hint that the inhibition of Wnt3a by Sclerostin follows a different mechanism than Wnt1 (Fig. 3A). However, the Sclerostin-neutralizing Fab antibody AbD09097 [41] rescues Sclerostin-mediated Wnt3a inhibition (Fig. 3B). Together with the variants ΔLoop and Alaloop of Sclerostin showing a 2.6- and 4.5-fold lower efficiency to antagonize Wnt3a (IC_50_ ΔLoop 655±143 nM, IC_50_ Alaloop 1114±475 nM, Fig. 3C) these observations suggest that the inhibition of Wnt3a by Sclerostin is specific. Thus although Sclerostin is possibly not a physiological inhibitor of Wnt3a in contrast to the classical Wnt antagonist Dkk1, it is mechanistically highly interesting how Sclerostin can interfere with LRP6 activation by Wnt3a. Despite the small differences for Wnt3a inhibition observed between wildtype Sclerostin and the loop-variants ΔLoop and Alaloop we analyzed the effect of our set of Sclerostin variants on Wnt3a activity using our reporter cell line. For almost all single amino acid variants the changes in the IC_50_ values were too small and thus statistically not significant (Fig. 3E). Even for the variant N92A, which showed a 200-fold decreased Wnt1 inhibition no differences could be observed compared to the wildtype protein. Interestingly, two mutants, the single residue variant R118A (located in Finger 2) and the multi-variant F2mut with an altered Finger 2 showed a small but statistically significant increase in the IC_50_ values compared to wildtype Sclerostin (IC_50_ R118A 606±74 nM; IC_50_ F2mut 682±177 nM, Fig. 3E, Fig. S1B). As alteration in the Finger 2 of Sclerostin showed no effect on Wnt1 inhibition this observation presents a further hint that mechanistically Sclerostin interferes with Wnt1 and Wnt3a activity by a different mechanism.

To get further insights into the different inhibition mechanism we analyzed the intracellular concentration of β-Catenin. A hallmark of canonical Wnt signaling is the accumulation of β-Catenin in the cytoplasm of the cell, where in the absence of Wnt factors a complex consisting of Axin, GSK3 and APC phosphorylates β-Catenin leading to its proteasomal degradation [46]. When we investigated β-Catenin degradation by quantitatively measuring the β-Catenin level in cells using a published assay [31], β-Catenin was decreased in a dose dependent manner by Sclerostin in cells stimulated with Wnt1 as expected and consistent with the findings of the reporter-gene analysis (Fig. 3A and D). However, when we analyzed Sclerostin-treated cells stimulated with Wnt3a, the β-Catenin level first remains constant and then even rises with high concentrations of Sclerostin (Fig. 3D), although these Sclerostin concentrations clearly attenuated the luciferase reporter-gene expression in our reporter cell line utilizing a β-Catenin dependent promoter (see also Fig. 3A). To test whether this surprising result might be specifically related to Wnt3a, we tested the classical Wnt antagonist Dkk1, which was shown to bind simultaneously to the first two and the last two propeller domains of LRP6 and thus can competitively inhibit Wnt factors of the Wnt1 as well as the Wnt3a subclass [44]. In contrast to Sclerostin, Dickkopf1 decreases intracellular β-Catenin levels in a dose dependent manner independent of the Wnt factor used for stimulation and thus shows that a competitive Wnt inhibition mechanism leads to a cytoplasmic decrease in β-Catenin as expected (Fig. 3D). Thus Sclerostin and Dickkopf1 clearly antagonize Wnt3a through a differing mechanism, how Sclerostin can decrease gene transcription driven from a β-Catenin promoter (see Fig. 3A) when it does not alter β-Catenin levels has to be subject for further studies.

### Binding of Sclerostin to LRP6 is usually required for Wnt inhibition

The Wnt-inhibiting activity of Sclerostin has previously been linked to its ability to bind to LRP co-receptors [26], [39]. As reduced LRP-binding of Sclerostin loop mutants was so far shown exclusively by in vitro interaction analysis using the isolated binding partners we wanted to examine the binding properties of our variant proteins in their cellular surrounding. We therefore performed a competition binding experiment using COS1 cells overexpressing human LRP6 and incubating them with ^125^I-labeled wildtype Sclerostin in presence or absence of unlabeled mutant proteins. The Sclerostin proteins were then crosslinked to the co-receptor LRP6 using disuccinimidyl suberate (DSS) and bis(sulfosuccinimidyl) suberate (BS^3^) and cell lysates were subsequently separated by SDS-PAGE. Without addition of an excess of unlabeled wildtype protein to compete off the radiolabeled Sclerostin from its receptor LRP6 we observed a prominent band in the autoradiography. This band was only visible in cells transfected with human LRP6 and therefore clearly corresponds to wildtype Sclerostin crosslinked to the extracellular domain of LRP6 (Fig. 4A, marked w/o). This band diminished when an excess (100-fold) of unlabeled wildtype Sclerostin was added (Fig. 4A, marked WT). Most Sclerostin variants showed competition efficiency similar to wildtype protein, except for the Sclerostin variants Alaloop and ΔLoop (Fig. 4A), as can also be seen in a summary showing the normalized results of several experiments (Fig. 4B). Since the latter mutants did not compete with the radiolabeled wildtype protein, they either do not bind at all or only with greatly diminished affinity to the extracellular domain of LRP6. To exclude the possibility that the radiolabeling interferes with the ability for competition, we repeated the experiment but using radiolabeled variant proteins. Iodination of Sclerostin ΔLoop and Alaloop showed that both loop variants do indeed not bind to LRP6 with high affinity as even with no competitor present only a faint band of the crosslinked product could be observed (Fig. S2A). Thus for the two Sclerostin loop variants the loss of LRP6 binding directly correlates with their lack of inhibiting Wnt1 or Wnt3a in the reporter gene assay and underlines the importance of the loop for Sclerostin activity and binding to LRP6. Consistently, also the single amino acid mutation of the loop residues Asp92 or Ile94 to alanine had a significant effect on binding to LRP6 albeit slightly smaller than what was observed for the variants ΔLoop and Alaloop (Fig. 4A,B). So possibly the slightly higher affinity for LRP6 of the two single residue mutants resulted in the stronger inhibition of Wnt3a activity (Fig. 3E), whereas they could not completely inhibit Wnt1 activity (Fig. 2A). Thus so far all loop mutants that were tested and show decreased affinity for LRP6 also exhibit a lower Wnt1 inhibition efficiency in line with the hypothesis that LRP6 binding and Wnt inhibition is correlated in Sclerostin.

This hypothesis seems also true for the variant C84AC142R affecting the cystine-knot. For the interaction analysis recombinant LRP6E1E2 protein was incubated with purified Sclerostin or variant proteins that were biotinylated in a 1∶1.5 stoichiometry using an NHS-activated biotin. After pull-down of the Sclerostin proteins via Streptavidin agarose the Sclerostin:LRP6 complex was probed with an antibody against the hexahistidine tag, which are present at the N-terminus of Sclerostin and at the C-terminus of LRP6E1E2 (Fig. 4C). Consistent with the radioligand binding assay wildtype Sclerostin is bound to LRP6E1E2, whereas the variant ΔLoop lacking residues Leu90 to Asn103 does not bind LRP6. For the cysteine variant C84AC142R, the Western Blot reveals a faint, but at longer exposure times clearly visible band thereby indicating that mutation of the third disulfide bond and introducing an arginine residue for Cys142 weakens binding to LRP6 (Fig. 4C, Fig. S2B). This suggests that the cystine-knot is likely also part of the Sclerostin epitope for binding to LRP6 and that the LRP5/6 binding site on Sclerostin contains additional elements in addition to the NXI-motif observed by Bourhis et al. [27]. Furthermore, all Sclerostin mutations that were tested and showed an attenuated inhibition of Wnt1 also exhibit decreased binding affinity for LRP6 indicating that LRP6 binding and Wnt1 inhibition is likely directly related.

However, in the analysis of Sclerostin residues affecting inhibition of Wnt3a two more mutants were identified, the Sclerostin variant R118A and the multi-variant F2mut (Fig. 3E, Fig. S1B). Both mutants show a significant reduction in the ability to inhibit Wnt3a-mediated reporter-gene expression, but do not affect inhibition of Wnt1 activity. As the residues exchanged in both variants are located in Finger 2 of Sclerostin and are thus structurally far remote from the LRP6 main binding determinant the NXI-motif (Fig. 1), it was unclear whether elements in Finger 2 play a role in LRP binding. Using the radioligand-binding assay on whole cells, the ability of these two mutants to compete with wildtype radiolabeled Sclerostin was tested. As both variants clearly replace the radiolabeled wildtype Sclerostin from its complex with LRP6 their binding to LRP6 seems unaltered (Fig. 4A,B; Fig. S2A). This presents a further hint that Sclerostin-mediated inhibition of Wnt3a occurs via an unknown non-competitive mechanism.

## Discussion

The role and impact of Wnt signaling in cancer is long known since the discovery that the integration of the mouse mammary tumor virus (MMTV) into the Wnt1a locus (int1) is the driver of virus-induced oncogenesis [47]. Besides its role in cancer biology Wnt signaling has gained great medical interest due to its involvement in bone homeostasis, initially discovered through gain- or loss-of-function mutations in the Wnt co-receptor LRP5 leading to either a high-bone mass phenotype or low bone density [36], [38], [48]. In this context, the Wnt inhibitors of the Dickkopf family and Sclerostin have received special attention, as both of them lead to bone loss by negatively regulating Wnt activity. Sclerostin was discovered from genetic screenings of patients showing increased bone densities, revealing that patients suffering from van Buchem disease or Sclerosteosis lack or have decreased levels of Sclerostin [14], [19]. Sclerostin's exclusive expression in bone by osteocytes [18] and the absence of any other phenotype other than increased bone density in the Sclerostin knockout mice [49] make Sclerostin a perfect target for osteoanabolic treatments. This led to a number of recent studies utilizing Sclerostin-neutralizing antibodies in animals [50], [51], [52] and humans [53], [54] showing that neutralization of Sclerostin can restore bone density in osteoporosis models [50] or other conditions resulting in bone loss [55], [56], [57], [58], [59].

**Figure 5:**
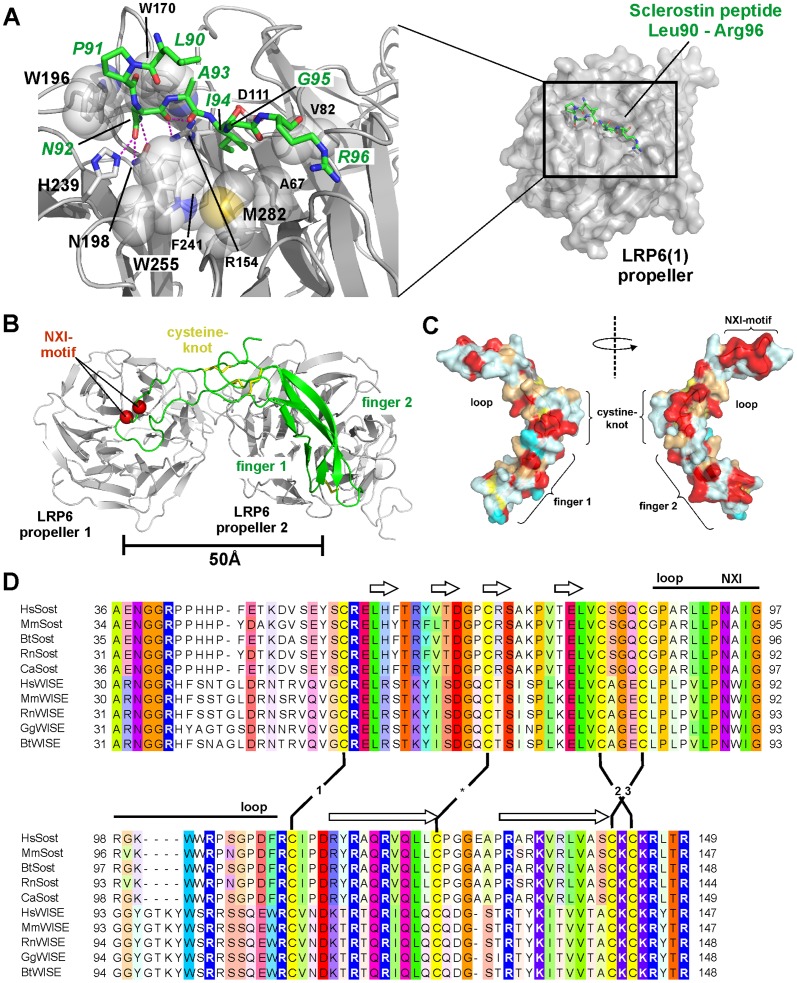
Modeling of a Sclerostin-LRP6E1E2 complex indicates additional interactions apart from the NXI motif. (A) Interaction scheme of a 7 mer peptide representing the loop tip amino acid sequence of Sclerostin (residues Leu90 to Arg96) bound into the cleft of the β-propeller domain 1 of LRP6 (PDB entry 3SOV, [27]). Two main binding determinants were identified in the Sclerostin-derived peptide, Asn92 and Ile94, which are engaged in several polar bonds (Asn92) as well as hydrophobic interactions (Ile94) to facilitate recognition and binding. Residues of LRP6 involved in the interaction with Asn92 and Ile94 are shown in the magnification on the left. (B) Theoretical model of the structured core domain of Sclerostin docked onto the propeller 1-2 fragment of LRP6. The two cavities formed by propeller domain 1 and 2 are separated by 50 Å. The extended architecture of Sclerostin (coincidentally measuring also 50 Å in length) might therefore contact propeller domain 1 and 2 (the latter with parts of Sclerostin finger 1 or 2) possibly explaining the higher binding affinity of Sclerostin to LRP6 fragments containing both propeller domains compared to LRP6 propeller 1 alone. (C) Surface representation of murine Sclerostin (left and right presentation are rotated by 180° around the y-axis) color-coded on the basis of an amino acid sequence alignment (D). The colors mark the level of amino acid identity/homology between Sclerostin and Wise proteins of different organisms with red highlighting invariant residues, orange for exchanges by homologous amino acids, and light and dark blue marking variable residue positions. (D) Sequence alignment of the core domain of Sclerostin and the related Wnt inhibitor Wise from different species. Secondary structure, architectural elements and disulfide bond pattern (1,2,3 mark the cystine-knot forming disulfides) are indicated.

Mechanistically, Sclerostin as well as members of the Dickkopf family inhibit canonical Wnt/β-Catenin signaling by binding to the Wnt co-receptors LRP5 or LRP6, however the binding sites on the large extracellular domain differ. Structural and functional studies revealed that the modular two-domain Dkk1 binds to the first and third of the four propeller domains of LRP5/6 (on the basis of the four propeller domains full-length extracellular LRP6 will be termed LRP6E1E4)) [26], [27], [44], [60], [61], [62], [63]. In contrast Sclerostin binds only to the first or the first two propeller domains of LRP6 (termed LRP6E1E2) as no measurable interaction was detected to a LRP6 deletion fragment comprising only propeller 3 and 4 (termed LRP6E3E4) [26], [27]. Consistently, mutations leading to a high-bone mass (HBM) phenotype and located in the first propeller domain of LRP5 only disrupt binding to Sclerostin whereas binding of Dkk1 seems unaffected [39]. Recent structure analyses of LRP6•Dkk1 interactions support this view. Here, the C-terminal cysteine-rich domain of Dkk1 engages tightly with an LRP6E3E4 fragment [60], [62], whereas a short peptide derived from the Dkk1 N-terminus (residues Asn7 to Asn13 of mature Dkk1) binds to the propeller 1 of LRP6 [27] (see also Fig. 5A). This explains the binding cooperativity seen in interaction analyses utilizing full-length or fragments of Dkk1 and LRP6. Full-length Dkk1 binds to the LRP6 fragments LRP6E1E2 and LRP6E3E4 with an affinity of 50 to 70 nM, whereas binding to full-length LRP6 occurs with very high affinity [44], [60].

In contrast to the data for Dkk1, structural analysis of the binding of Sclerostin to propeller 1 of LRP6 is so far limited to a short 7 mer peptide sequence homologous between Dkk1 and Sclerostin sharing a so-called NXI-motif (Fig. 5A) [27]. In Sclerostin the NXI-motif is located in the tip of the dynamic loop. Analyses of Bourhis et al. and Holdsworth et al. have so far identified two main binding determinants for binding of Sclerostin to LRP6 [27], [43]. One, the conserved asparagine residue engages in five hydrogen bonds, three of which emanate from its carboxamide side chain (Fig. 5A) [27]. Two further hydrogen bonds involve the backbone carbonyl of the NXI-asparagine. The corresponding co-receptor residues are invariant in LRP5/6 and are part of the HBM mutations [27]. The other determinant of the NXI-motif, the isoleucine residue, deeply immerses into a hydrophobic pocket built by various conserved residues in LRP6, which again are either part of the HBM mutations or are in close proximity to known HBM mutations (Fig. 5A) [27]. Noteworthy, almost all the co-receptor key residues are also conserved in LRP4, which has been identified as a co-receptor for Sclerostin and Dkk1 [64]. Furthermore, most interacting residues of LRP6 are also found in LDLR, which does not bind Sclerostin [25]. However, whereas coordination of the NXI-asparagine might be accomplished as in LRP5/6, the hydrophobic cleft for the NXI-isoleucine residue differs between LRP and LDLR suggesting that both residues in the motif are equally important for recognition of the Wnt modulator proteins (Fig. S3).

Most importantly, the small epitope of the 7 mer peptide in the LRP6-peptide complex seems insufficient to account for the high-affinity interaction of Sclerostin to LRP6, which was determined at about 10 nM for the binding to LRPE1E4 or LRP6E1E2 [27]. Indeed, the large excess of peptides required to crystallize the LRP6-peptide complexes [27] and the low inhibition efficiency (IC_50_ between 10 to 30 µM) for 7 mer peptides comprising the NXI-motif [27], [43] indicate that additional interactions between full-length Sclerostin and LRP6 exist. A detailed analysis of Sclerostin binding to individual LRP6 propeller domains revealed an affinity of about 60 nM for the first and a binding constant of 1.3 µM to the second propeller domain of LRP6 [27] suggesting that elements outside the NXI-motif likely interact with propeller 2. However, the analyses of Holdsworth et al. and Veverka et al. did not identify additional residues around the NXI-motif that strongly influence Sclerostin-mediated inhibition of Wnt1 activity [40], [43]. The larger loss of Wnt1 inhibition observed for our Sclerostin multi-variants ΔLoop and Alaloop, which contain a truncated or scrambled loop region (Leu90 to Asn103), compared to single point mutants N92A and I94A (multi-variants showed a 4-times higher IC_50_ value) might result from additional small contributions of residues close by the NXI motif or elsewhere in the loop. Also, the remaining Wnt inhibition capabilities of the loop variants ΔLoop and Alaloop (see Fig. 2C) are a clear indication for additional, albeit weak interactions outside the investigated loop segment of Sclerostin and the LRP co-receptors. A simple and naïve model of a Sclerostin-LRP6E1E2 complex can be build by first docking the structures of free Sclerostin [28], [40] and the complex of the Sclerostin peptide bound to LRP6E1 [27] and then replacing the structure of the single propeller domain by a structure of LRP6E1E2 [62]. Due to the elongated Sclerostin architecture - the finger tips and the loop span about 45 to 55 Å - contacts between Sclerostin and both propeller domains of LRP6E1E2 are easily possible as the pockets of both propellers are also 50 to 55 Å apart from each other (Fig. 5B). The high flexibility and the various conformations of the loop region as seen in the NMR structures of Sclerostin make this model rather preliminary and don't provide conclusive ideas about additional epitopes [28], [40]. But given the large diameter of the LRP propeller 1 and the extended conformation the Sclerostin loop can adopt, it seems a reasonable assumption that the flexible loop and/or the central region of Sclerostin share additional contact(s) with the rim of the LRP6 propeller 1 in a fashion similar to what is seen in structure of the extended Laminin E1 module bound to the propeller of Nidogen [65].

Our study now indeed identified the cystin-knot as a potentially LRP6-interacting element in Sclerostin. Although the original mutation C142R found in a Turkish patient suffering from Sclerosteosis possibly acts through a gene-dosage effect resulting from a diminished secretion of the variant protein [42], we were interested whether this mutation might also have a direct functional impact on LRP binding and Sclerostin's Wnt inhibition capabilities. Instead of using the original mutation we employed the double variant C84AC142R to avoid low protein yield or misfolding caused by the free thiol group of the unpaired cysteine. The Sclerostin variant, which lacks the third disulfide of the cystine-knot and instead has a bulky charged side chain at position 142, exhibits a strongly decreased inhibition efficiency for Wnt1 although not as strong as the Sclerostin loop variants ΔLoop and Alaloop. A pull-down analysis then showed that the binding of Sclerostin C84AC142R to the ectodomain of LRP6 is indeed impaired strongly indicating that Cys142 and/or the region around the third disulfide bond of the cystine-knot is a second epitope for LRP6 interaction. A further hint that parts outside the NXI-motif are likely involved in LRP6 binding comes from the NMR titration study performed by Holdsworth et al. [43]. The authors showed that many chemical shifts of the residues located in Sclerostin's structured core are affected by binding to the LPR6E1E2 fragment, which is due to the fact that the Sclerostin core now exhibits the slow overall tumbling rate of the large LRP6E1E2•Sclerostin complex. Only the residues in the flexible N- and C-termini of Sclerostin do not show massive line broadening, as their motions within the highly dynamic termini is not restricted. If only residues in the dynamic loop, i.e. the NXI-motif, would be involved in LRP6E1E2 binding all NMR signals from residues in Finger 1 and 2 of Sclerostin should still be observable (a.k.a. show no line broadening) as was shown for the NMR mapping study employing a Sclerostin-neutralizing Fab antibody fragment [40]. A final point might come from sequence analysis of Sclerostin and the related Wnt-inhibitor Wise [66], which shows high sequence homology also outside the NXI-motif and mapping onto the Sclerostin structure indeed indicates a highly similar protein surface starting from the loop tip down to finger 2 (Fig. 5C,D).

A mechanistically interesting aspect in our study is that the different Sclerostin-mediated inhibition of the two Wnt factors Wnt1 and Wnt3a, both of which are reported to signal via the canonical Wnt/β-Catenin pathway, differs [67], [68]. The teams of Rami Hannoush and Mike Costa convincingly showed that the different Wnt factors bind to different propeller domains of LRP6 [44], [69]. Wnt3/Wnt3a bind to propellers 3–4, but not to the LRP6E1E2 fragment, in contrast, Wnt9b binds to the first two propeller domains but not to the LRP6E3E4 fragment showing that these two Wnt factors utilize distinct and non-overlapping epitopes on LRP6 [44]. Using neutralizing anti-LRP6 antibodies specifically raised against LRP6E1E2 and LRP6E3E4 the canonical Wnt factors could be grouped into two functional groups with respect to their binding site on LRPs, with Wnt3/3a employing propellers 3–4 and Wnt1/2/2a/6/8a/9a/9b/10b interacting exclusively with propeller domains 1 and 2 [69]. Thus Dkk1, which was shown to bind to the LRP6 propeller domains 1 and 3 can efficiently antagonize both Wnt groups via a competitive binding inhibition mechanism [67], [68]. For Sclerostin, however, whose binding site is limited to the propeller 1 and 2 of LRPs [25], [27], one would assume that inhibition by this particular Wnt modulator is limited to the group Wnt1/2/2a/6/8a/9a/9b/10b, but the non-overlapping binding sites for Sclerostin and Wnt3/Wnt3a should result in no inhibition at all against Wnt3/Wnt3a. Conformingly, in our setup Sclerostin neutralizes Wnt1 activity with a low IC_50_ of about 10 nM, which nicely correlates with the reported binding affinity of Sclerostin to the LRP6 extracellular domain [27]. The dose-response curve for the inhibition of Wnt1 by increasing concentrations of Sclerostin yields a Hill-coefficient consistent with a competitive binding of Wnt1 and Sclerostin to LRP6. Remarkably, Sclerostin is capable to also neutralize Wnt3a activity, however, a half-maximal concentration (IC_50_) of about 200 to 250 nM of Sclerostin is required. Since a more than 20-fold higher Sclerostin concentration is necessary to antagonize Wnt3a in comparison to Wnt1, this might question whether Sclerostin is a physiologically relevant Wnt3a inhibitor in vivo. Mechanistically interesting, analysis of the dose-response curves revealed a Hill coefficient for Wnt3a inhibition of five or even larger (see Fig. 3A), which is characteristic for a non-competitive mechanism [70]. As other studies have also shown that Sclerostin can inhibit Wnt3a in vitro [24], [26], [28], [40] we have to accept that Sclerostin is (at least from a mechanistic point of view) a Wnt3a inhibitor even though Sclerostin and Wnt3a do not exhibit overlapping binding sites on LRP6 [26], [27] and thus the inhibition cannot be explained by a pure competition mechanism. In line with a different inhibition mechanism for Wnt1 and Wnt3a our functional study also reveals differences in Sclerostin inhibiting either Wnt1 or Wnt3a activity. Although the loop region comprising residues Leu90 to Asn103 seems important for neutralizing Wnt activity of both Wnt factors, the effects of other mutations in Sclerostin differed between Wnt1 and Wnt3a. While residues outside the loop except for Cys142 did not influence inhibition of Wnt1 activity, mutations in finger 2, variants F2mut and R118A (see also Fig. 3E; Fig. S1B) altered Wnt3a inhibition without affecting binding to LRP6, which is consistent with a non-competitive mechanism. Finally, a direct hint that the mechanism by which Sclerostin inhibits Wnt1 and Wnt3a differs mechanistically is seen from the measurements of cytoplasmic β-Catenin levels via in cell Western analysis. The SuperTopFlash reporter construct used in numerous reports to study canonical Wnt/β-Catenin signaling [34] measures β-Catenin translocation into the nucleus via expression of the reporter gene luciferase, which is under the control of a TCF/LEF-promoter. In the absence of Wnt, β-Catenin is phosphorylated by a complex comprising GSK3, Axin and APC and is destined for proteasomal degradation. In the presence of Wnt phosphorylation of β-Catenin is abolished and the accumulating β-Catenin is translocating from the cytoplasm into the nucleus where it acts as transcriptional coactivator. Thus a dose-dependent decrease in expression of the luciferase reporter upon inhibition of either Wnt1 or Wnt3a by a Wnt antagonist protein suggests an identical underlying mechanism for both Wnt factors leading to proteasomal degradation of cytoplasmic β-Catenin and thus a subsequent lack of transcriptional activation due to lower β-Catenin levels in the nucleus. For the Wnt modulator Dkk1 this holds true as the decrease in the reporter gene measurements correlates with a decrease in β-Catenin concentration (see also Fig. 3D). However, in the case of Sclerostin the level of intracellular β-Catenin were only decreased, when Wnt1 was antagonized by Sclerostin (similar to Dkk1), but for Wnt3a, inhibition by Sclerostin even led to an increase in cytoplasmic β-Catenin. Thus, whereas Sclerostin-mediated down-regulation of Wnt1 activity is indeed due to degradation of β-Catenin, inhibition of Wnt3a activity by Sclerostin is probably caused by an impaired or blocked nuclear import of β-Catenin.

In summary, these observations point towards a more complex regulatory mechanism by which Sclerostin modulates the activities of different Wnt factors. A molecular scheme by which the Wnt antagonists Dkk1 and Sclerostin inhibit activation of the β-Catenin canonical signaling pathway exclusively by competing for the shared receptor LRP5/6 does not sufficiently explain all inhibitory activities of Sclerostin. There have been reports about Wnt-induced LRP aggregation/oligomerization [71], [72] as possible part of Wnt receptor activation and antibody-mediated artificial dimerization of LRP6 potentiated Wnt activity [69]. The antagonist-LRP interaction could besides directly competing with the Wnt-LRP interaction alter this oligomerization and thus modulate the signaling state of the LRP co-receptor. The structure analyses of the Dkk1 C-terminal domain bound to LRP6E3E4 revealed a second interaction site, which is too large to be solely explained by crystal-lattice contacts and leads to a dimerized LRP6 [60], [62]. The physiological significance of this interaction is yet unclear, as mutagenesis of residues in this second interface did not alter the Dkk1-mediated inhibition of Wnt3a activity in a Wnt reporter assay [62]. However, this might be misleading, as Sclerostin also showed inhibition of Wnt1 and Wnt3a in a similar Wnt reporter assay, but analysis of the β-Catenin levels clearly suggest a different mechanism. Finally, low-resolution structure studies of the full-length extracellular domain of LRP6 showing the ectodomain as a dynamic architecture with the propellers 1–2 and 3–4 connected by a flexible hinge were already used to explain simultaneous binding of full-length Dkk1 to propellers 1 and 3 [61], [62]. Modeling suggests that the LRP ectodomain can adopt an arc-like curvature [43] bringing the first and the last two propeller domains in close proximity thereby also bringing Dkk1 and Sclerostin into close proximity with all domains of LRP5/6 when bound (Fig. S4). Thus activation by Wnt factors as well as their inhibition by Sclerostin or Dkk family members might involve an allosteric conformational switch in the LRP ectodomain as suggested [71], [72], [73] possibly explaining how Sclerostin can inhibit Wnt3a without sharing common binding epitopes on LRP6. The data suggest that activation and inhibition of the canonical Wnt/β-Catenin pathway is much more complex and requires further structural and functional analyses.

## Supporting Information

Figure S1 **Surface plasmon resonance analysis using a conformation sensitive Fab-fragment and additional reporter gene data of Sclerostin mutants.** (A) A sensogram of the interaction of the antibody AbD10723 and either Sclerostin WT or Sclerostin ΔLoop, the latter of which were immobilized on the surface of a ProteOn™ GLC sensor chip, is shown. At time point zero the antibody was perfused for 200 seconds. Dissociation was initiated by injecting only running buffer and monitoring the dissociation for 250 seconds. Binding affinities (K_D_) obtained by fitting each binding curve using a Langmuir type 1∶1 interaction model (ProteOn™ Manager 3.0 software (BioRad)) are indicated. (B) Reporter gene assay employing HEK293TSA cells stably transfected with the Wnt-responsible luciferase reporter construct SuperTOPFlash and stimulated with 1.5 nM recombinant mWnt3a and serial dilutions of Sclerostin WT or the indicated variants.
(TIF)

Figure S2 **Binding of Sclerostin variants to LRP6 in radioligand binding and pulldown assays.** (A) COS1-cells were transfected with hLRP6 and were incubated with I^125^-labeled Sclerostin variants (I^125^). Cell-bound protein was chemically crosslinked in the presence of different amounts of unlabeled wildtype Sclerostin or unlabeled Sclerostin finger 2 multi-variant F2mut (Comp. x fold) and analyzed by autoradiography. (B) Longer exposure time for the Western Blot of pull down experiment using the biotinylated Sclerostin mutants C84AC142R, ΔLoop and WT Sclerostin (compare Fig. 4C).
(TIF)

Figure S3 **Sequence alignment of the N-terminal propeller domain of LRP5 and 6 (LRP5E1 and LRP6E1) with the equivalent propeller domains of LRP4 and LDLR.** Whereas LRP4, 5, and 6 are known to bind Sclerostin, LDLR was shown to not interact with the Wnt inhibitor. The sequence alignment reveals that the asparagine residue of the NXI motif observed in the LRP6E1:Sclerostin peptide structure (PDB entry 3SOV, [27]) can be similarly accommodated as the structural environment in the propeller domains is identical (contact residues are indicated by NXI with bold letter for N). However, contact residues for the interaction with the isoleucine residue of the NXI motif differ (marked by NXI with bold letter I), in particular the hydrophobic residues Trp255 and Met282 are replaced by smaller or charged residues (Ile602 and Glu629, respectively) thereby likely preventing binding of the Sclerostin NXI motif to LDLR.
(TIF)

Figure S4 **Modeling suggests an arc like curvature of the LRP6 ectodomaine.** (A) Model of the extracellular domain of LRP6 comprising all four propeller domains (LRP6E1E4). The model was built by assembling the four propeller architecture by structurally super-imposing the first propeller of one LRP6E1E2 moiety (molecule 1) (PDB entry 3S94) onto the second propeller domain of a second LRP6E1E2 moiety (molecule 2) (for assembly see B). This intermediate overlay was then used to structurally align propeller 3 of a LRP6E3E4 fragment (PDB entry 3S8Z) onto the second propeller of the realigned LRP6E1E2 molecule (molecule 1) and connecting the LPR6E1E2 (molecule 2) and the LRP6E3E4 fragment to provide the curved architecture for the extracellular domain of LRP6E1E4. As the linker sequences between each of the propeller domains share a similar length and amino acid composition (see sequence alignment) the arc-like architecture observed in low resolution structure studies [61], [62] is likely a consequence of the propeller 2 and 3 adopting a similar interaction/conformation as observed in the LRP6 fragment structures 1–2 and 3–4, which have been considered to form rather rigid 2-propeller architectures. Analysis of the model consisting of the full-length extracellular domain and particular the modeled linker region between propeller 2 and 3 using rigid body analysis, e.g. PiSQRD, http://pisqrd.escience-lab.org and HingeProt, http://bioinfo3d.cs.tau.ac.il/HingeProt/have not revealed a different mobility for the three linker regions. Thus conformational rearrangement of the LRP6 architecture might be due to individual rigid body movements of either one of the four propeller domains. The curved assembly observed in the low-resolution studies might be stabilized by similar hydrogen bond connections between the individual propellers. The “inter-propeller” hydrogen bonds observed (C, E) or predicted (D) are shown with the linker region at the end of one EGF-like domain to the beginning of the next YWTD domain marked in red.
(TIF)
