# Correction: Mutational Analysis of Sclerostin Shows Importance of the Flexible Loop and the Cystine-Knot for Wnt-Signaling Inhibition

**DOI:** 10.1371/annotation/067f1197-3290-4d1e-8f77-4d3996a6b9e8

**Published:** 2014-01-06

**Authors:** Verena Boschert, Maarten van Dinther, Stella Weidauer, Katharina van Pee, Eva-Maria Muth, Peter ten Dijke, Thomas D. Mueller

Supporting Information file S2 is a duplicate of file S1. Please see the correct Figure S2 here: http://plosone.org/corrections/pone.0081710.s002.cn.tif

