# Supplementary figures and images for: Mutational Analysis of Sclerostin Shows Importance of the Flexible Loop and the Cystine-Knot for Wnt-Signaling Inhibition
Source: PLoS One. 2013 Nov 29;8(11):e81710. doi: 10.1371/journal.pone.0081710 (PMC3843708; doi:10.1371/journal.pone.0081710)

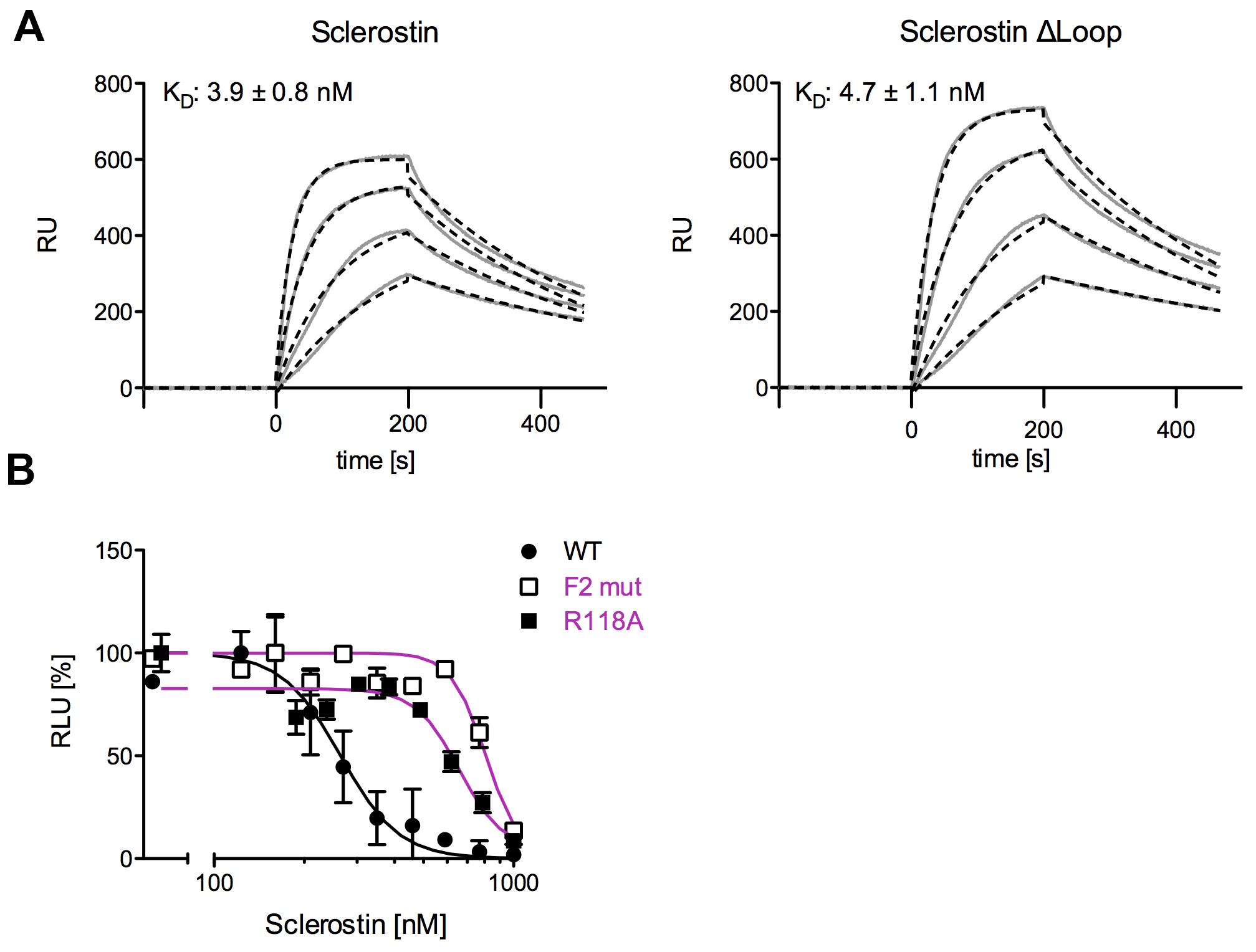

Supplement: Figure S1 — Surface plasmon resonance analysis using a conformation sensitive Fab-fragment and additional reporter gene data of Sclerostin mutants. (A) A sensogram of the interaction of the antibody AbD10723 and either Sclerostin WT or Sclerostin ΔLoop, the latter of which were immobilized on the surface of a ProteOn™ GLC sensor chip, is shown. At time point zero the antibody was perfused for 200 seconds. Dissociation was initiated by injecting only running buffer and monitoring the dissociation for 250 seconds. Binding affinities (KD) obtained by fitting each binding curve using a Langmuir type 1∶1 interaction model (ProteOn™ Manager 3.0 software (BioRad)) are indicated. (B) Reporter gene assay employing HEK293TSA cells stably transfected with the Wnt-responsible luciferase reporter construct SuperTOPFlash and stimulated with 1.5 nM recombinant mWnt3a and serial dilutions of Sclerostin WT or the indicated variants. (TIF) [file pone.0081710.s001.tif]

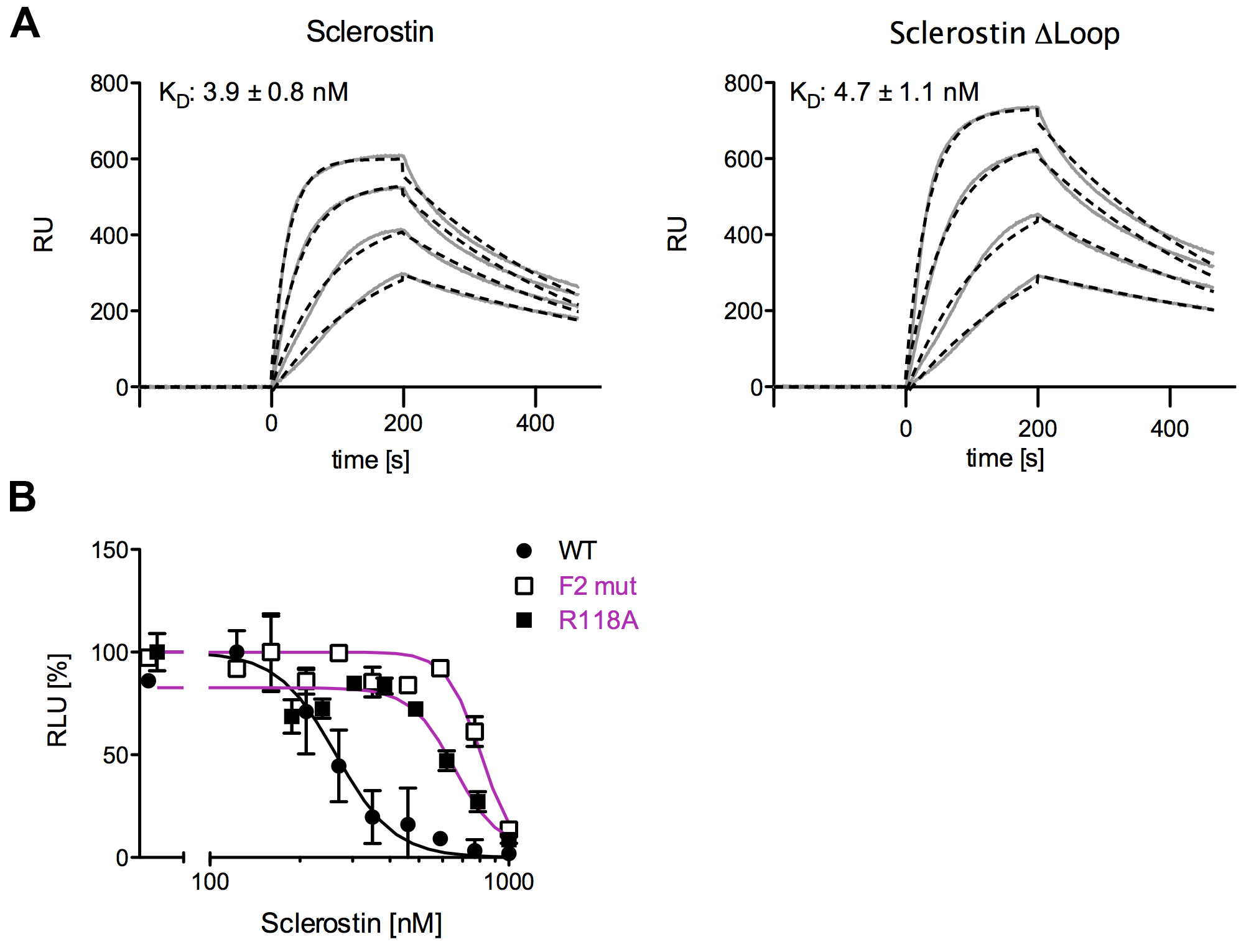

Supplement: Figure S2 — Binding of Sclerostin variants to LRP6 in radioligand binding and pulldown assays. (A) COS1-cells were transfected with hLRP6 and were incubated with I125-labeled Sclerostin variants (I125). Cell-bound protein was chemically crosslinked in the presence of different amounts of unlabeled wildtype Sclerostin or unlabeled Sclerostin finger 2 multi-variant F2mut (Comp. x fold) and analyzed by autoradiography. (B) Longer exposure time for the Western Blot of pull down experiment using the biotinylated Sclerostin mutants C84AC142R, ΔLoop and WT Sclerostin (compare Fig. 4C). (TIF) [file pone.0081710.s002.tif]

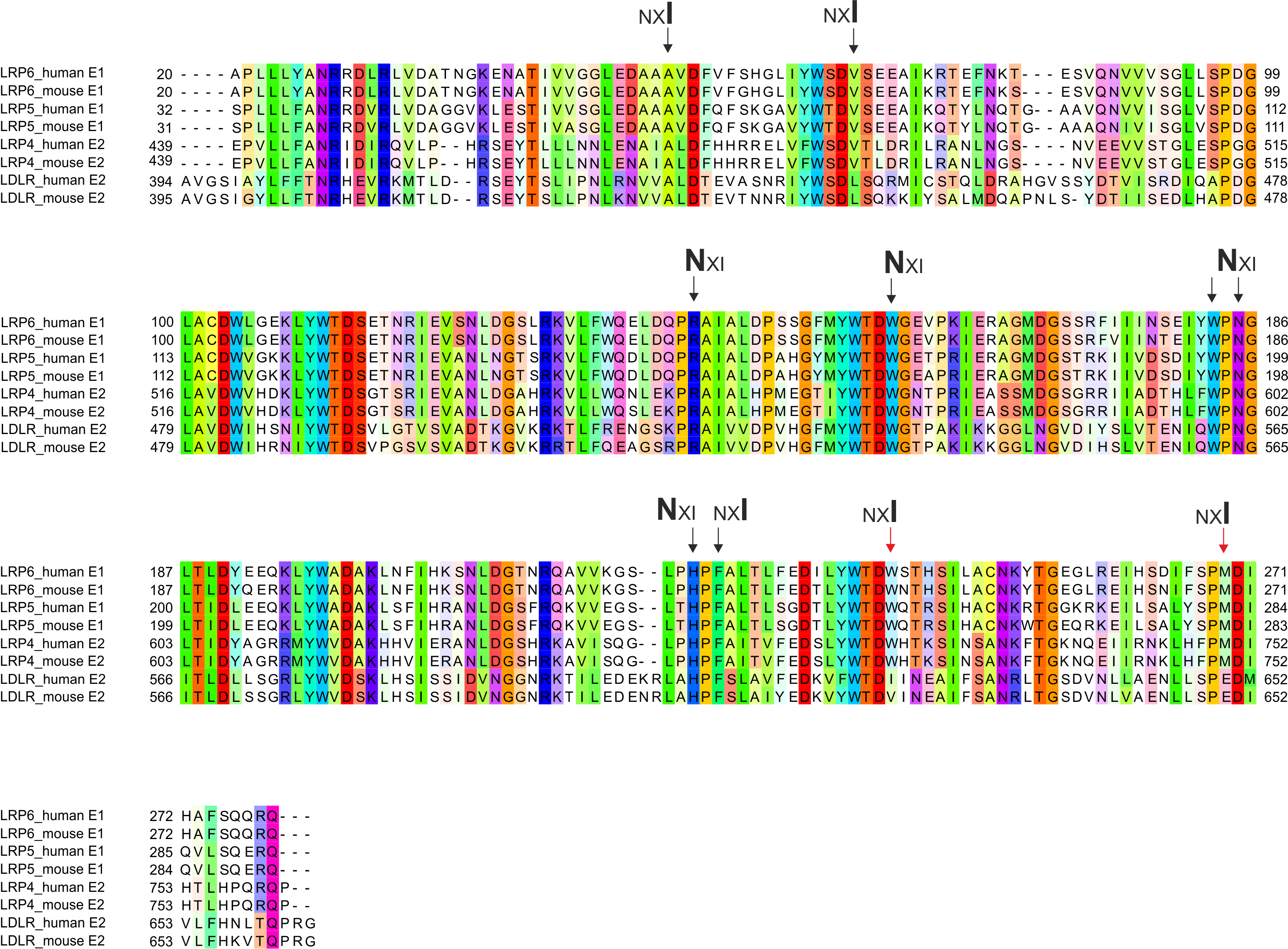

Supplement: Figure S3 — Sequence alignment of the N-terminal propeller domain of LRP5 and 6 (LRP5E1 and LRP6E1) with the equivalent propeller domains of LRP4 and LDLR. Whereas LRP4, 5, and 6 are known to bind Sclerostin, LDLR was shown to not interact with the Wnt inhibitor. The sequence alignment reveals that the asparagine residue of the NXI motif observed in the LRP6E1:Sclerostin peptide structure (PDB entry 3SOV, [27]) can be similarly accommodated as the structural environment in the propeller domains is identical (contact residues are indicated by NXI with bold letter for N). However, contact residues for the interaction with the isoleucine residue of the NXI motif differ (marked by NXI with bold letter I), in particular the hydrophobic residues Trp255 and Met282 are replaced by smaller or charged residues (Ile602 and Glu629, respectively) thereby likely preventing binding of the Sclerostin NXI motif to LDLR. (TIF) [file pone.0081710.s003.tif]

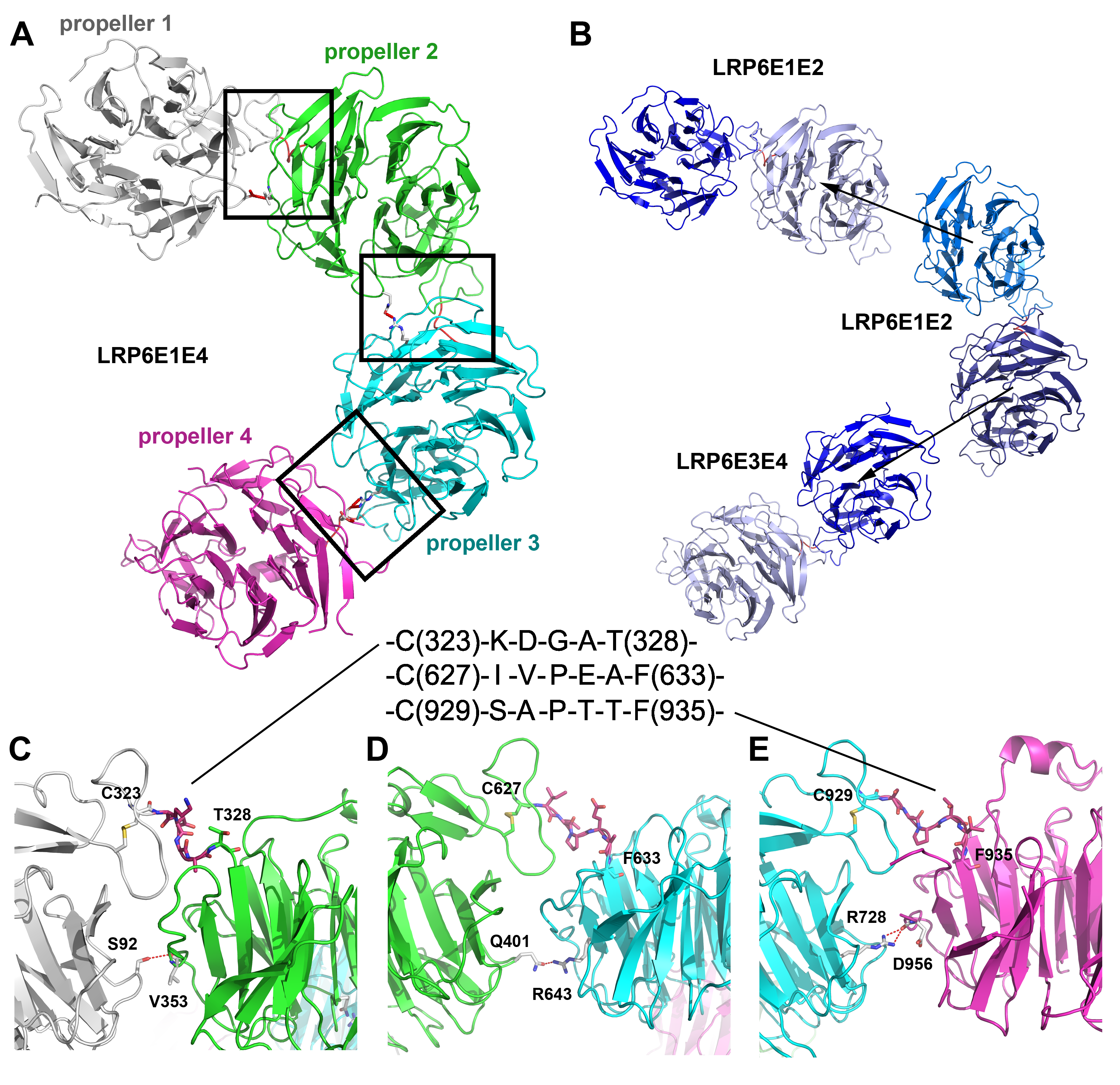

Supplement: Figure S4 — Modeling suggests an arc like curvature of the LRP6 ectodomaine. (A) Model of the extracellular domain of LRP6 comprising all four propeller domains (LRP6E1E4). The model was built by assembling the four propeller architecture by structurally super-imposing the first propeller of one LRP6E1E2 moiety (molecule 1) (PDB entry 3S94) onto the second propeller domain of a second LRP6E1E2 moiety (molecule 2) (for assembly see B). This intermediate overlay was then used to structurally align propeller 3 of a LRP6E3E4 fragment (PDB entry 3S8Z) onto the second propeller of the realigned LRP6E1E2 molecule (molecule 1) and connecting the LPR6E1E2 (molecule 2) and the LRP6E3E4 fragment to provide the curved architecture for the extracellular domain of LRP6E1E4. As the linker sequences between each of the propeller domains share a similar length and amino acid composition (see sequence alignment) the arc-like architecture observed in low resolution structure studies [61], [62] is likely a consequence of the propeller 2 and 3 adopting a similar interaction/conformation as observed in the LRP6 fragment structures 1–2 and 3–4, which have been considered to form rather rigid 2-propeller architectures. Analysis of the model consisting of the full-length extracellular domain and particular the modeled linker region between propeller 2 and 3 using rigid body analysis, e.g. PiSQRD, http://pisqrd.escience-lab.org and HingeProt, http://bioinfo3d.cs.tau.ac.il/HingeProt/have not revealed a different mobility for the three linker regions. Thus conformational rearrangement of the LRP6 architecture might be due to individual rigid body movements of either one of the four propeller domains. The curved assembly observed in the low-resolution studies might be stabilized by similar hydrogen bond connections between the individual propellers. The “inter-propeller” hydrogen bonds observed (C, E) or predicted (D) are shown with the linker region at the end of one EGF-like domain to the beginning of th [file pone.0081710.s004.tif]
